# First molecular detection and genetic diversity of *Hepatozoon* sp. (Apicomplexa) and *Brugia* sp. (Nematoda) in a crocodile monitor in Nakhon Pathom, Thailand

**DOI:** 10.1038/s41598-024-54276-6

**Published:** 2024-02-12

**Authors:** Witchuta Junsiri, Patchana Kamkong, Piyanan Taweethavonsawat

**Affiliations:** 1 Parasitology Unit, Department of Veterinary Pathology, Faculty of Veterinary Science, Chulalongkorn University, Bangkok, 10330 Thailand; 2 Biomarkers in Animals Parasitology Research Unit, Chulalongkorn University, Bangkok, 10330 Thailand

**Keywords:** Genetics, Microbiology, Molecular biology

## Abstract

The crocodile monitor (*Varanus salvator*) is the most common monitor lizard in Thailand. Based on habitat and food, they have the potential to transmit zoonoses, with a high possibility of infecting ectoparasites and endoparasites. Diseases that could infect crocodile monitors and be transmitted to other animals, including humans. This research aims to identify and evaluate the phylogenetic relationships of *Hepatozoon* sp. and sheathed microfilaria in crocodile monitors. The phylogenetic analyses of *Hepatozoon*, based on 18S rRNA, and sheathed microfilaria, based on the COX1 gene, revealed that the *Hepatozoon* sp. were grouped with *H. caimani,* while sheathed microfilaria were grouped together with *B. timori*. This study provides insights into the genetic diversity and host-parasite interactions of hemoparasites in crocodile monitors in Thailand.

## Introduction

Crocodile monitors, which belong to the Varanidae family, are categorized into a singular genus called *Varanus*^1,2^. They can be found in various geological locations such as mainland and islands, spanning across Africa, Central Asia, the Middle East, the Arabian Peninsula, the Indo-Australian Archipelago, and South and Southeast Asia, which includes Thailand^1,3–6^. Since 1992, the crocodile monitor, known as *V. salvator*, has been classified as a “reserved wild animal” and listed in the Act of Animals Protection and Conservation of Thailand. They are predatory creatures that can be found in freshwater wetlands and urban waterways across the country. Limited research has been conducted to examine the microbial ecology of crocodile monitors, their role as hosts or reservoirs for pathogens transmitted by arthropods, and their interactions with ectoparasites^3,4,7^. However, parasitic infections in *Varanus* spp. have been investigated in Australia, Nigeria, Slovenia, South Africa, and Thailand^8–12^. *Hepatozoon* is a prevalent blood parasite species commonly found in the Asian crocodile monitor and various other reptiles. In South Africa, a prevalence of 25% was observed^12^, while Brazil ranged from 1.1 to 12.5%^13,14^, Iran had a prevalence of 39.72%^15^ and Australia had a high prevalence of 58.1%^16^.

Onchocercidae, Dirofilariinae, and *Oswaldofilaria* sp. have been reported in the abdominal cavity and pleural, peritoneal, and lung nodules of *Varanus bengalensis* (*V. bengalensis*)*,* identified using a traditional blood smear preparation^9^. Nevertheless, there is a lack of comprehensive research on the crocodile monitor, specifically regarding the identification of parasites at the molecular level. In Thailand, only a single study has been conducted, which examined *Hepatozoon* sp. gamonts and reported their presence in less than 1% of the red blood cells (RBCs) of 43 crocodile monitors^17^. Hence, the objective of this study was to assess the phylogenetic distribution of hemogregarine and filarial nematodes in crocodile monitors from Thailand by comparing them to documented parasitic species found in diverse hosts and geographical regions.

## Results

### Morphological and morphometric analysis

**Figure 1:**
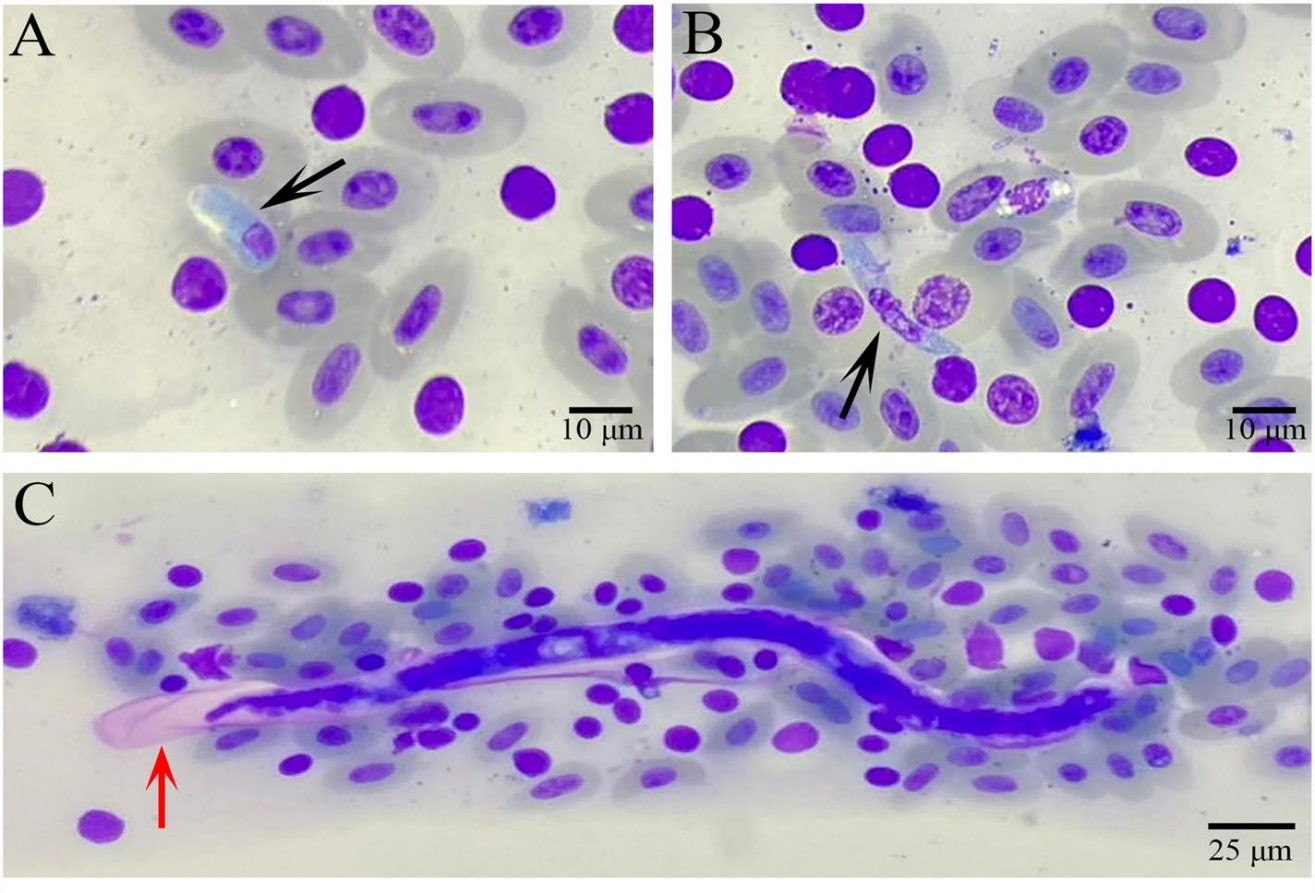
Giemsa staining of hemogragarine gamonts and sheathed microfilaria infections from crocodile monitors in Thailand. Black arrows indicate the presence of hemogregarine gamonts of *Hepatozoon* (**A**, **B**). Red arrows indicate the presence of sheathed microfilaria (**C**).

Out of the two free-living crocodile monitor specimens screened, one was found to be infected with *Hepatozoon* spp. and a filarial nematode, as shown in Fig. 1. The morphological and morphometric data analysis allowed identification of one morphotype of *Hepatozoon* spp. However, it was not possible to determine the species of the morphotype, and thus, it was classified as an undescribed species. The examination of blood smears revealed the presence of mature gamonts, as depicted in Figs. 1A,B.

In the blood smear, only mature gamonts were identified. These mature gamonts were found within a parasitophorous vacuole (PV) and had a rounded shape at both ends. The cytoplasm of the gamonts displayed a bluish-purple stain. The elongated nucleus exhibited purple-stained chromatin and occupied nearly half of the surface area of the parasite. Gamonts measured (mean ± standard deviation) 11.32 ± 0.886 in length and 4.25 ± 0.621 in width (n = 30) (Table 1).

**Table 1:** The target genes and primers (sequence and length) used to detect and characterize crocodile monitor parasites.

Parasitic infection	Target gene	Primers	Sequence (5′–3′)	Annealing temperature (°C)	Product size (bp)	References
Hemogregarines	18S rRNA	HepF300	GTTTCTGACCTATCAGCTTTCGACG	60	600	^20^
		HepR900	CAAATCTAAGAATTTCACCTCTGAC			
		HEMO1	TATTGGTTTTAAGAACTAATTTTATGATTG	48	900	^21^
		HEMO2	CTTCTCCTTCCTTTAAGTGATAAGGTTCAC			
Microfilariae	18S rRNA	Pan-Nem-18SF	TCGTCATTGCTGCGGTTAAA	54	1127–1155	^40^
		Pan-Nem-18SR	GGTTCAAGCCACTGCGATTAA			
	COX1	Pan-Fil cox1F	ATRGTTTATCAGTCTTTTTTTATTGG	52	650	^41^
		Pan-Fil cox1R	GCAATYCAAATAGAAGCAAAAGT			
	12S rRNA	Pan-Fil 12SF	TCCAGAATAATCGGCTATACATTTT	56	497–570	^42^
		Pan-Fil 12SR	CCATTGACGGATGGTTTGTA			

### Molecular amplification, sequencing, and similarity of *Hepatozoon* 18S rRNA and *Brugia* COX1 sequences

One captured crocodile monitor was observed with both hemogregarine and filarial infections (Fig. 1A,B). The polymerase chain reaction (PCR) products were sequenced for the 18S rRNA gene (hemogregarine) and COX1 (filarial). Three genes were amplified for filarial nematodes: the 18S rRNA, COX1, and 12S rRNA genes. Unfortunately, only the COX1 sequence was successfully amplified. All PCR amplicons were sequenced in both directions. Sequences were deposited in GenBank under the accession numbers OQ306503 (*Hepatozoon* sp. 18S rRNA gene sequence) and OQ338200 (*Brugia* sp.COX1 sequence). The basic local alignment search tool (BLAST) result for the 18S rRNA *Hepatozoon* sequence (917 base pairs [bps], Supplementary Fig. 1A) indicated 99% identity with *Hepatozoon* sp. from *Philodryas patagoniensis* (MN003368, Uruguay) and *Tarentola deserti* (KU680460, Morocco) and 98% identity with *H. caimani* (KU495923, Brazil) from caiman crocodiles. The COX1 sequence (651 bps, Supplementary Fig. 1B) demonstrated 96% identity with *B. timori* (AP017686), which was previously deposited in GenBank. The Thailand *Hepatozoon* sp. 18S rRNA sequence demonstrated 99.9% similarity with *H. caimani* (KU495923, Brazil), as shown in Table 2. The *Brugia* sp. COX1 sequence had 96.9% similarity with *B. timori* (AP017686, Japan) and 95.4% with *B. timori* (KP760173, Indonesia), as shown in Table 3.

**Table 2:** The similarity of the *Hepatozoon* 18S rRNA gene sequence from a crocodile monitor in Thailand compared with global sequences.

Acc. No	Host	Haemogregarines	Similarity (%)												
OQ306503_Thailand	*Varanus salvator*	*Hepatozoon* sp.	100												
KU495923_Brazil	*Caiman crocodilus yacare*	*H. caimani*	99.9	100											
KJ413113_Brazil	*Caiman crocodilus yacare*	*Hepatozoon* sp.	98.5	98.6	100										
KJ413115_Brazil	*Caiman crocodilus yacare*	*Hepatozoon* sp.	98.2	98.3	98.7	100									
HQ734807_Morocco	*Timon pater tangitana*	*Hepatozoon* sp.	99.0	99.1	98.2	98.5	100								
MK508984_Brazil	*Leptodactylus latrans*	*Hepatozoon* sp.	98.9	99.0	98.8	98.6	99.2	100							
MN003357_Uruguay	*Philodryas patagoniensis*	*Hepatozoon* sp.	98.6	98.7	97.1	98.1	97.7	99.8	100						
KM234615_Brazil	*Hemidactylus mabouia*	*Hepatozoon* sp.	98.7	98.8	98.1	98.2	98.5	99.3	98.0	100					
MZ412879_Iran	*Macrovipera lebetina obtusa*	*Hepatozoon* sp.	97.4	97.5	98.1	97.5	98.5	97.8	96.9	98.2	100				
HQ734787_Algeria	*Tarentola mauritanica*	*Hepatozoon* sp.	98.8	99.0	98.4	98.1	98.4	99.0	97.5	98.5	98.5	100			
HQ292771_Seychelles	*Mabuya wrightii*	*Hepatozoon* sp.	98.8	99.0	98.5	98.1	98.5	98.9	97.4	98.6	98.6	99.8	100		
KJ574012_Egypt	*Cerastes cerastes*	*Hepatozoon* sp.	98.0	98.1	99.2	99.0	99.4	98.2	98.6	99.3	97.1	99.0	98.9	100	
KF939622_China	*Elaphe carinata*	*Hepatozoon* sp.	98.2	98.3	98.3	97.9	98.2	98.4	97.3	98.3	97.7	98.5	98.6	98.1	100
KU680459_Algeria	*Tarentola deserti*	*Hepatozoon* sp.	98.5	98.6	98.7	98.4	98.5	98.9	97.7	98.5	98.1	99.0	99.0	98.9	99.2
KC696566_Portugal	*Psammophis aegyptius*	*Hepatozoon* sp.	98.7	98.8	98.9	98.5	98.8	99.0	97.6	98.7	98.7	99.1	99.2	99.9	99.5
MG249965_India	*Ptyas mucosa*	*Hepatozoon* sp.	98.4	98.5	98.4	98.1	99.1	98.5	98.2	99.1	97.2	99.0	99.0	98.2	98.7
KM234647_Madagascar	*Madagascarophis colubrinus*	*Hepatozoon* sp.	99.0	99.1	98.9	98.6	98.7	99.3	97.7	98.7	98.8	99.2	99.3	99.8	99.4
JX644998_Hungary	*Myodes glareolus*	*Hepatozoon* sp.	98.2	98.3	98.5	98.2	98.2	98.5	97.0	98.2	97.9	98.4	98.5	99.0	98.6
AY600625_Spain	*Clethrionomys glareolus*	*Hepatozoon* sp.	98.1	98.2	98.3	98.1	98.1	98.4	97.0	98.1	97.7	98.3	98.4	98.1	98.5
OM033660_Brazil	*Akodon* sp.	*Hepatozoon* sp.	98.3	98.5	98.3	98.1	98.2	98.6	97.3	98.3	98.2	98.7	98.8	99.3	98.9
FJ719818_Chile	*Abrothrix olivaceus*	*Hepatozoon* sp.	98.8	98.7	98.6	98.3	98.5	98.8	97.3	98.3	98.3	98.7	98.8	99.7	99.1
AB181504_Thailand	*Bandicota indica*	*Hepatozoon* sp.	97.7	97.6	97.9	97.6	97.7	98.2	96.9	97.9	97.6	98.1	98.2	97.8	98.4
OM033665_Brazil	*Decomys mamorae*	*Hepatozoon* sp.	97.9	98.0	98.1	97.7	97.9	98.3	96.8	97.7	97.7	98.1	98.2	98.6	98.4
MK452252_Canada	*Sciurus carolinensis*	*H. griseisciuri*	98.4	98.5	98.5	98.3	98.3	98.9	97.7	98.5	98.0	98.8	98.8	98.2	98.9
MH198742_Saudi Arabia	*Spalerosophis diadema*	*H. aegypti*	98.8	98.9	97.8	98.5	97.7	99.1	97.0	97.6	97.5	98.0	98.1	99.5	98.5
MN723845_Iran	*Pseudopus apodus*	*H. ophisauri*	98.8	98.9	98.8	98.5	98.7	99.1	97.7	98.7	98.5	99.1	99.1	99.9	99.5
KM234649_Madagascar	*Furcifer* sp.	*H. domerguei*	99.1	99.2	98.7	98.4	98.7	99.1	97.7	98.8	98.8	99.3	99.3	99.3	98.8

Acc. No	Similarity (%)														
OQ306503_Thailand															
KU495923_Brazil															
KJ413113_Brazil															
KJ413115_Brazil															
HQ734807_Morocco															
MK508984_Brazil															
MN003357_Uruguay															
KM234615_Brazil															
MZ412879_Iran															
HQ734787_Algeria															
HQ292771_Seychelles															
KJ574012_Egypt															
KF939622_China															
KU680459_Algeria	100														
KC696566_Portugal	99.8	100													
MG249965_India	98.9	99.8	100												
KM234647_Madagascar	99.7	99.9	99.2	100											
JX644998_Hungary	99.0	99.4	98.8	99.3	100										
AY600625_Spain	98.8	99.3	98.7	99.2	99.5	100									
OM033660_Brazil	99.3	99.5	98.9	99.4	99.0	98.9	100								
FJ719818_Chile	99.6	99.7	99.1	99.6	99.1	99.1	99.4	100							
AB181504_Thailand	98.7	99.1	98.2	99.0	98.4	98.5	98.9	99.0	100						
OM033665_Brazil	98.9	99.1	98.2	99.0	98.4	98.5	98.9	99.0	98.4	100					
MK452252_Canada	99.2	99.5	98.8	99.5	98.9	98.9	99.1	99.3	98.9	98.7	100				
MH198742_Saudi Arabia	98.9	99.0	99.3	98.9	98.3	98.2	98.4	98.7	97.9	97.9	98.4	100			
MN723845_Iran	99.8	100	99.3	99.9	99.2	99.1	99.4	99.6	98.9	98.9	99.4	99.0	100		
KM234649_Madagascar	99.1	99.3	98.8	99.5	98.6	98.6	98.8	98.9	98.3	98.3	98.9	98.3	99.3	100

**Table 3:** The similarity of the sheathed microfilaria COX1 gene sequence from a crocodile monitor in Thailand compared with global sequences.

Acc. no	Host	Haemogregarines	Similarity (%)																				
OQ338200_Thailand	*Varanus salvator*	*Brugia* sp.	100																				
AP017686_Japan	Unknown	*B. timori*	96.9	100																			
KP760173_Indonesia	*Homo sapiens*	*B. timori*	95.4	100	100																		
AP017680_Japan	Unknown	*B. pahangi*	90.1	92.1	91.6	100																	
MT027204_Thailand	Unknown	*B. pahangi*	89.7	92.1	92.1	98.7	100																
NC004298_USA	Unknown	*B. malayi*	89.5	92.3	92.8	90.3	90.6	100															
MT149211_Thailand	*Homo sapiens*	*B. malayi*	89.7	92.4	93.0	90.4	90.8	99.8	100														
OP265710_Canada	*Canis lupus familiaris*	*Brugia* sp.	89.5	92.3	92.1	90.3	90.3	89.9	90.1	100													
MT193074_French Guiana	*Canis lupus familiaris*	*Brugia* sp.	90.1	93.6	93.7	91.3	91.8	93.2	93.2	92.7	100												
NC016186_USA	Unknown	*W. bancrofti*	87.7	90.4	90.9	90.4	90.6	91.0	91.2	90.3	91.1	100											
AP017705_USA	Unknown	*W. bancrofti*	87.8	90.6	91.4	90.6	90.8	91.2	91.4	90.7	91.5	99.5	100										
LC318284_Japan	*Capricornis* sp.	*O. flexuosa*	84.4	87.3	88.8	85.5	86.9	87.5	87.5	87.6	88.0	87.0	87.4	100									
AB518875_Japan	*Simulium bidentatum*	*O. dewittei japonica*	86.5	88.2	88.8	88.0	87.8	87.3	87.5	88.4	90.8	88.6	89.0	89.0	100								
AJ271617_Cameroon	Unknown	*O. gutturosa*	85.9	89.3	88.0	88.5	88.1	88.1	88.3	88.3	88.0	89.3	89.7	90.9	90.3	100							
AM749268_Japan	*Cervus nippon*	*O. eberhardi*	86.2	88.6	89.2	85.7	85.7	86.4	86.4	88.7	88.9	87.4	87.8	90.5	92.1	91.3	100						
KP760203_Japan	*Sus scrofa leucomystax*	*O. japonica*	86.9	89.0	89.5	88.6	88.6	87.9	87.9	88.8	91.4	88.8	89.0	90.2	99.8	90.9	93.1	100					
ON641583_China	*Pyrrhocorax pyrrhocorax*	*Neofoleyellides* sp*.*	81.8	83.6	83.9	83.2	83.4	83.0	83.2	84.7	85.2	85.2	85.7	83.4	84.4	85.9	84.9	85.0	100				
OP040124_Australia	*Notama cropus agilis*	*Breinlia boltoni*	85.6	88.2	87.7	86.7	86.9	87.3	87.3	87.2	87.9	88.4	88.6	87.8	86.8	89.2	87.5	87.9	83.7	100			
KY085963_Argentina	*Canis lupus familiaris*	*Dirofilaria* sp.	86.0	88.5	88.5	87.7	87.5	88.7	88.7	89.7	89.4	88.5	89.1	90.0	91.4	91.9	91.9	92.7	85.1	87.5	100		
MK032318_USA	*Contra canadensis*	*D. lutrae*	84.3	86.4	86.1	86.7	87.2	86.1	86.1	88.4	87.4	88.1	88.5	88.7	89.8	90.6	89.8	90.0	84.6	88.0	92.5	100	
KX265050_India	*Homo sapiens*	*Dirofilaria* sp.	85.2	87.8	89.2	87.6	88.7	88.7	88.9	89.7	90.1	89.3	89.8	89.2	92.8	92.4	92.2	93.3	85.8	87.8	94.3	91.3	100

### Phylogenetic analysis of *Hepatozoon* 18S rRNA and *Brugia* COX1 sequences

**Figure 2:**
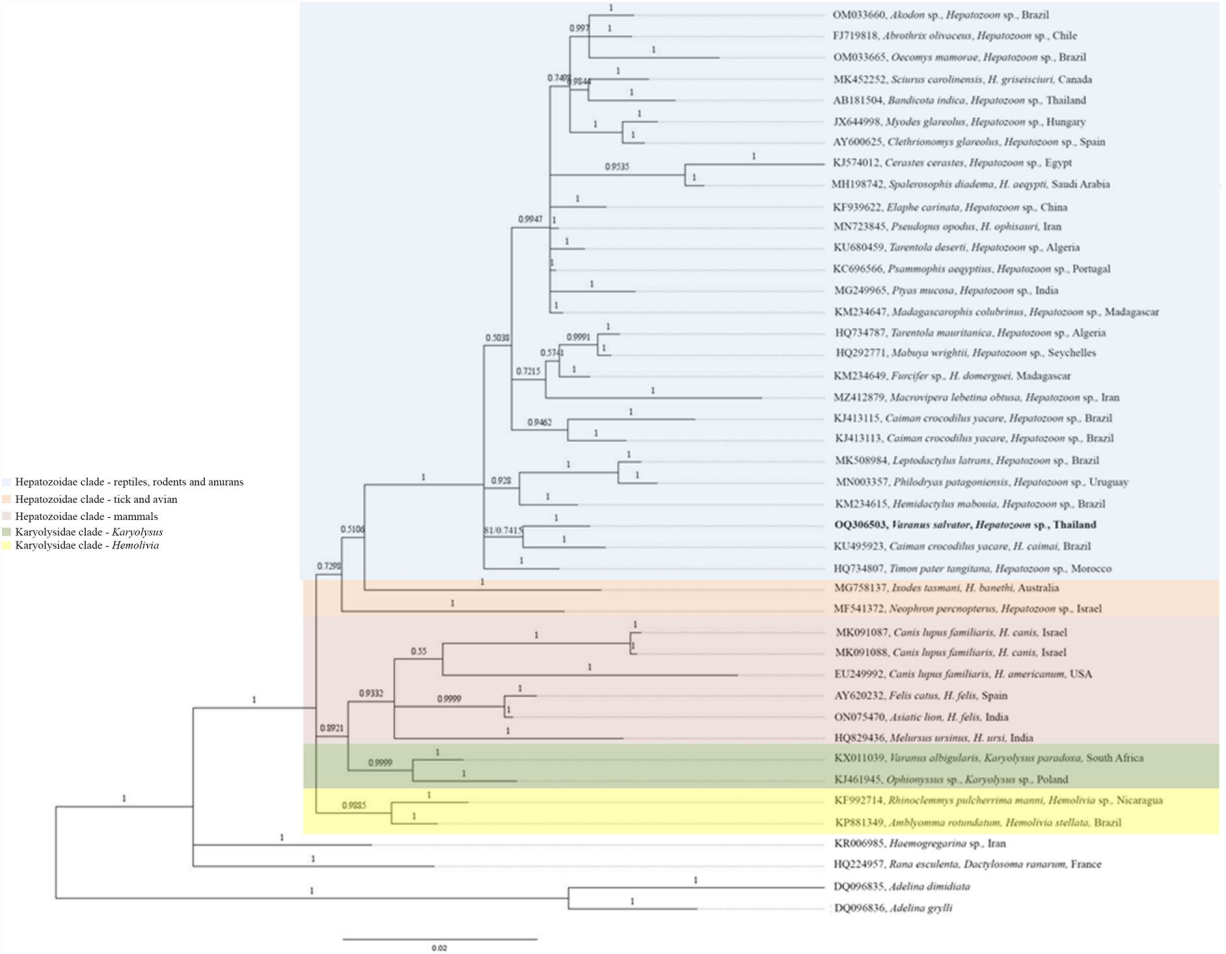
The phylogenetic tree constructed using the Maximum Likelihood (ML) and Bayesian inference (BI) method based on Hepatozoon 18S rRNA sequences (917 bp). The isolates *Adelina dimidiate* (DQ096835) and *Adelina grylli* (DQ096836) were used as an out-group and the sequence of this study is in bold.

**Figure 3:**
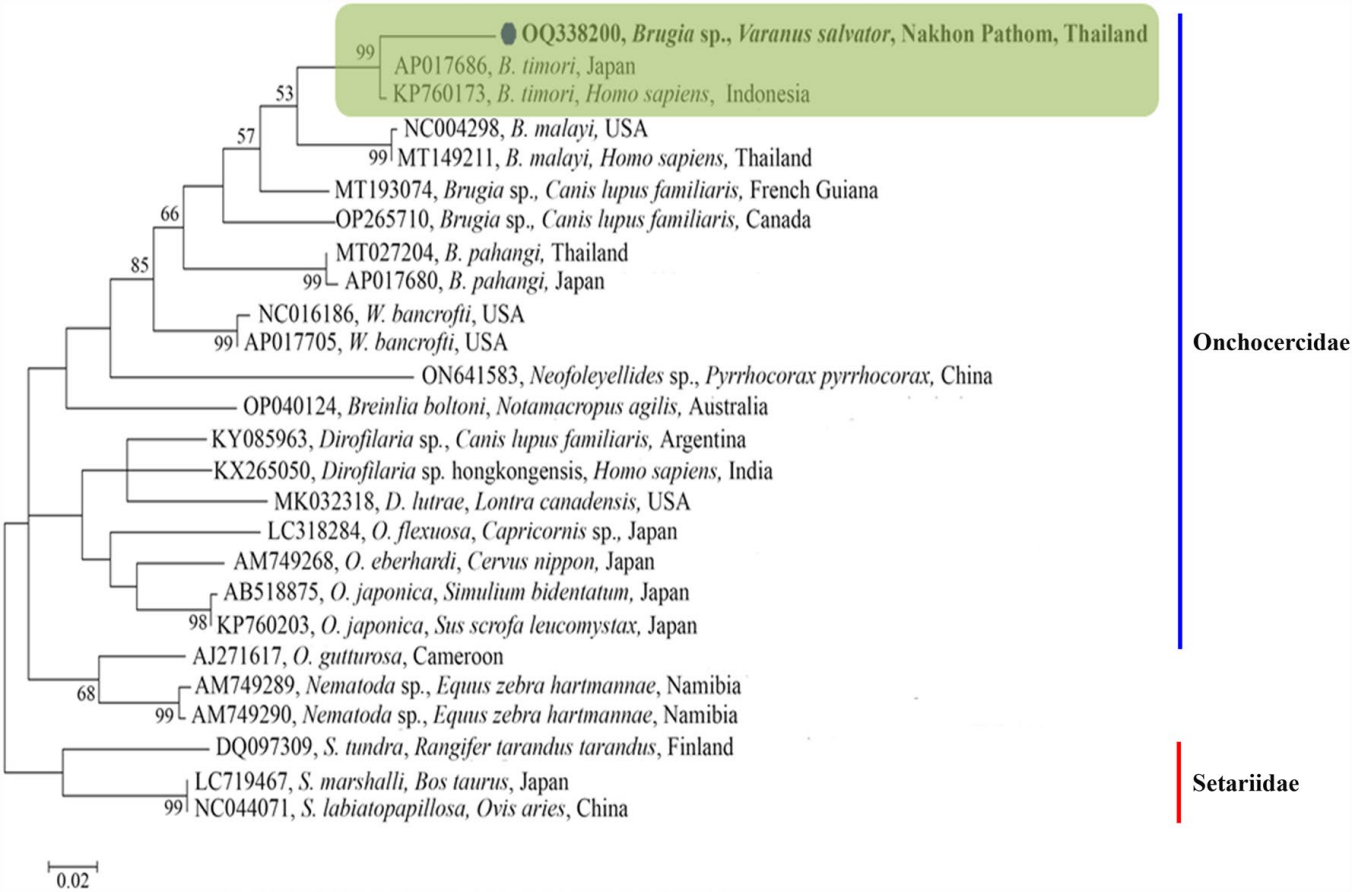
The phylogenetic tree constructed using the maximum likelihood method and sheathed microfilaria COX1 sequences. The bootstrap values are shown at branching points and the sequence of this study is in bold.

The phylogenetic tree of *Hepatozoon* sp. 18S rRNA gene sequences demonstrated clustering in a monophyletic group together with sequences of *H. caimani* (KU495923) that were recently detected in caiman crocodiles in Brazil (Fig. 2). The phylogenetic tree was comprised of eight branches. The first branch included the *Hepatozoon* sp. sequence amplified in the present study and sequences retrieved from GenBank from other reptile taxa (e.g., snakes, lizards, geckos, and caiman crocodiles), rodents, and amphibians. *Hepatozoon* sequences amplified from tick (MG758137) and vulture (MF541372) were grouped in the second and third branches, respectively. The forth branch included the *Hepatozoon* sequences received from mammals (e.g. dogs, cats, lions and bears). In addition, the remaining branches comprising species from *Karyolysus*, *Hemolivia*, *Haemogregarina* and *Dactylosoma*. sp. *Adelina dimidiate* (DQ096835) and *Adelina grylli* (DQ096836) were used as the out-group (Fig. 2). The phylogenetic tree of Onchocercidae COX1 sequences were clustered in a monophyletic group comprising *B. timori*, *B. malayi*, *Brugia* sp, *B. pahangi*, *W. bancrofti*, *Neofoleyellides* sp., *Breinlia boltoni*, *Dirofilaria* sp., *Dirofilaria* sp. “hongkongensis,” *D. lutrae*, *O. flexuosa*, *O. eberhardi*, and *O. japonica* (Fig. 3). The amplified filarial sequence was clustered with sequences of *B. timori* (AP017686 and KP760173), recently detected in humans in Japan and Indonesia. *Nematoda* sp. and *Setaria* sp. were used as the out-groups (Fig. 3). Moreover, the reliability of bootstrap frequencies and Bayesian posterior probabilities of all phylogenies are displayed with the highest values on each branch.

### Haplotype diversity

**Figure 4:**
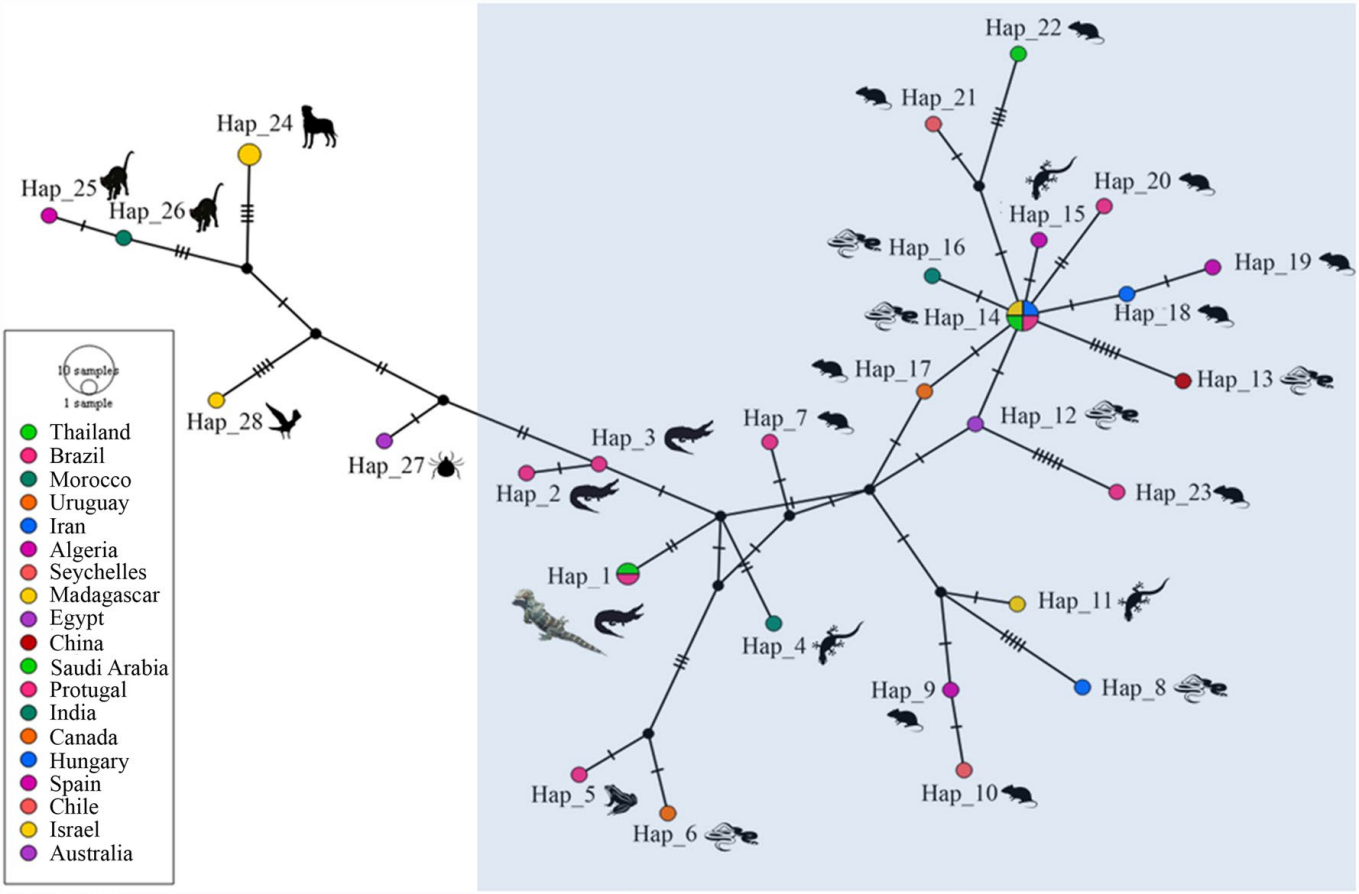
Templeton, Crandall, and Sing network of haplotypes based on the *Hepatozoon* 18S rRNA gene sequences examined in Thailand and globally. The small traits between haplotypes indicate the occurrence of mutations.

**Figure 5:**
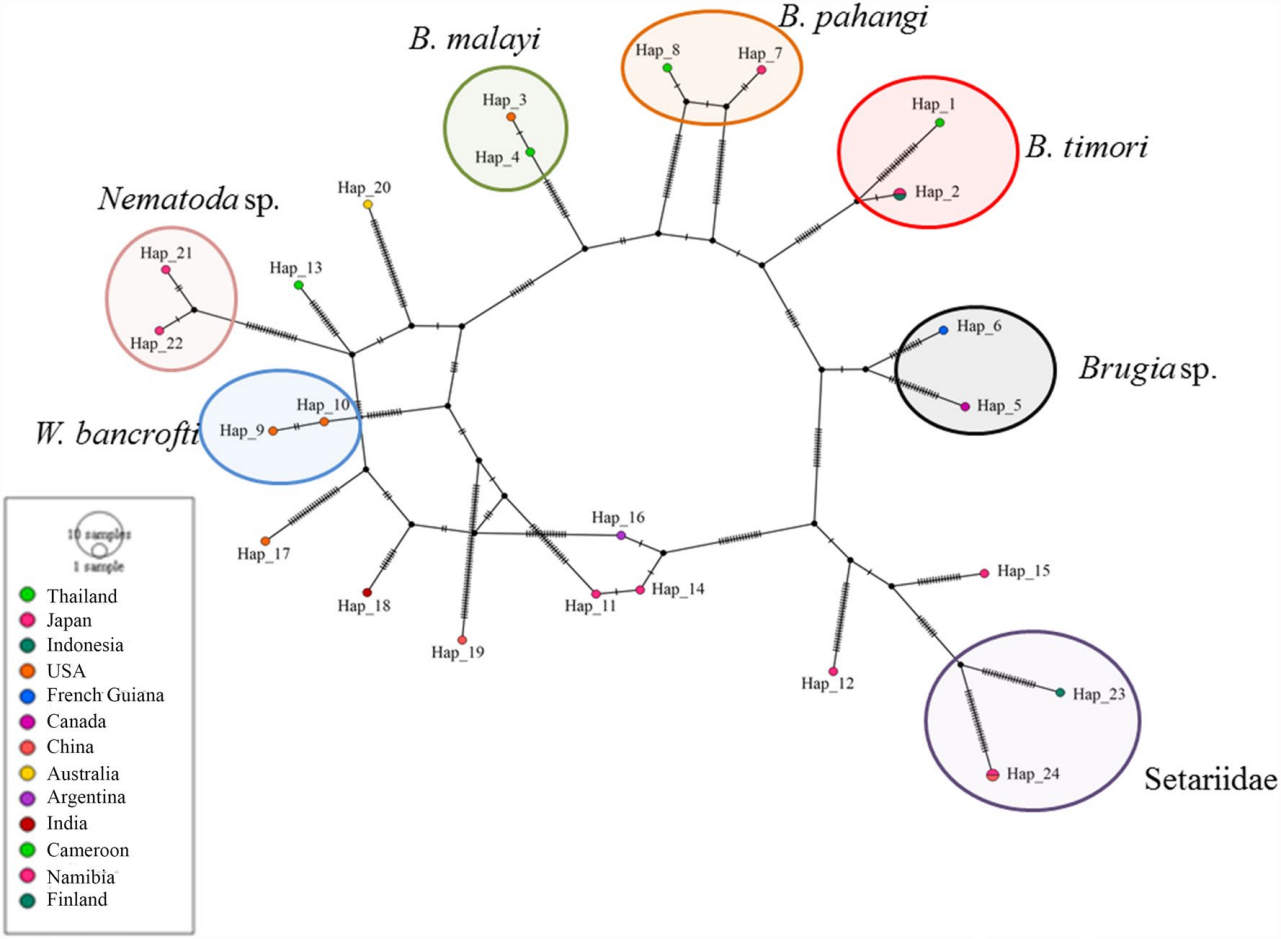
Templeton, Crandall, and Sing network of haplotypes based on the sheathed microfilaria COX1 gene sequences examined in Thailand and globally. The small traits between haplotypes indicate the occurrence of mutations.

Nucleotide polymorphisms and DNA divergence between the sequences obtained in the present study and GenBank sequences were analyzed. Nucleotide polymorphism analysis of *Hepatozoon* sp. 18S rRNA and *Brugia* sp. COX1 sequences revealed 28 and 24 haplotypes, respectively (Table 4). The haplotype networks of these genes were obtained from the Templeton, Crandall, and Sing (TCS) Network tool (Figs. 4, 5). For the *Hepatozoon* sp. 18S rRNA gene, of the 28 haplotypes, haplotype 1 was detected in the crocodile monitor from the Nakhon Pathom provinces and in *H. caimani* in the caiman crocodile from Brazil. Haplotypes 1–23 were found in reptiles, rodents, and amphibians, while the remaining haplotypes were found in tick, canine, feline, and avian hosts from a range of countries (Table 3, Fig. 4). The haplotype network of *Brugia* sp. COX1 gene demonstrated that the sequence from the Thailand crocodile monitor was detected in haplotype 1, while the *B. timori* sequences from Japan and Indonesia were detected in haplotype 2 (Table 3, Fig. 5).

**Table 4:** Polymorphisms and genetic diversity of *Hepatozoon* 18S rRNA and sheathed microfilaria COX1 sequences from a crocodile monitor in Thailand compared with global sequences.

Genes	Size (bp)	*N*	VS	GC%	h	Dh (mean ± SD)	π (mean ± SD)	*K*
*Hepatozoon* sp. 18S rRNA	950	33	57	42.7	28	0.985 ± 0.014	0.00937 ± 0.00102	7.71212
Sheathed microfilaria COX1	648	26	122	32.6	24	0.994 ± 0.013	0.11056 ± 0.00457	49.86154

*N* = number of analyzed sequences; VS = number of variable sites; GC = G × C content; h = nunber of haplotypes; Dh = diversity of haplotypes; SD = standard deviation; π = nucleotide diversity (per site);* K* = average number of nucleotide differences.

## Discussion

Until now, the detection of *Hepatozoon* infections in crocodile monitors has been only reported in Bangkok, Thailand, by means of microscopy screening of blood samples^17^. Morphologic and morphometric studies could enable differentiation between *Hepatozoon* sp.^18^ However; a single parameter cannot be used to differentiate species using the microscopic technique. The need for an additional tool, such as the molecular technique, is needed to enable this. In addition, there is an apparent lack of relevant information regarding the genetic diversity of *Hepatozoon* sp. and microfilaria isolated from crocodile monitors in Thailand. The current study is the first investigation on the phylogeny of *Hepatozoon* and sheathed microfilaria isolated from crocodile monitors in Nakhon Pathom, Thailand.

The findings of this study show that crocodile monitors from Thailand can harbor various parasites, including both hemogregarines and filarial worms. The phylogenetic results revealed that *Hepatozoon* sp. in crocodile monitors had 99% similarity with *H. caimani*, and the *Hepatozoon* 18S rRNA gene was grouped in the same clade as crocodiles, reptiles, rodents, and amphibians. In this case, it is not possible for us to make a definitive determination about how the transmission occurs. However, it is plausible that transmission could occur either through prey-predator interactions or via vectors. This has been observed in African reptiles^19^ and described in the case of *H. domerguei* infection in native reptiles from Madagascar^20^. Prey-predator transmission occurs when a predator ingests infectious cysts present in its prey. Additionally, to confirm vector-borne transmission, it is necessary to ascertain and identify the developmental stages of arthropod vectors.

In this study, the oligonucleotide pairs HepF300/900 and HEMO1/HEMO2 were used to amplify the *Hepatozoon* 18S rRNA gene^21,22^. This method had already been successful in inferring phylogenetic relationships between *Hepatozoon* spp. from snakes^23,24^. Oligonucleotides 18S and 5.8S have also been used to successfully infer phylogenetic relationships between *Hepatozoon* spp. from reptiles, amphibians and mammals^23–26^. The utilization of oligonucleotides HEMO1 and HEMO2 enabled the identification of a new species of *Hepatozoon* in *Coluber constrictor priapus* and *Thamnophis sauritus sackenii*. Through their application, the researchers were able to establish the phylogenetic relationship among hemogregarine isolates originating from Florida^27^. Therefore, the 18S rRNA sequence is useful for characterization and comparing phylogenetic relationships generic affiliations without prior knowledge of the sporogonic development of parasites^28^.

The phylogenetic results for the sheathed microfilaria COX1 gene in crocodile monitors revealed that it closely related to *B. timori* in humans from Japan and Indonesia. However, little is known about phylogenetic relationships of filarial worms in crocodile monitors. The phylogenetic relationship of filarial worms in wild endemic reptiles from Madagascar has been previously described^20^. In Malaysia, a new genus, *Malayfilaria*, along with a new species, *M. sofiani*, was identified in common tree shrews using the COX1 and 12S rRNA genes and the ITS1 region^29^. *M. sofiani* appears most closely related to *Wuchereria* spp. and *Brugia* spp.; however, it differs in several morphological characteristics. Therefore, it is important to assess the real prevalence of this parasite and investigate its implication for the host, as filarial nematodes, such as *B. malayi* and *B. timori*, are known to cause lymphatic diseases in humans living in tropical areas, while *B. pahangi* infects carnivores and causes zoonotic diseases in humans^29^. Unfortunately, in the current study, the PCRs targeting the 18S and the 12S rRNA genes failed to amplify sheathed microfilaria DNA. However, parasitemia levels may have been lower, thereby potentially resulting in the failure to detect parasites using PCR. Therefore, the infection levels reported in previous studies that used different primers should be compared with caution.

## Conclusions

This report presents the first findings on the molecular detection of *Hepatozoon* sp. 18S rRNA gene and sheathed microfilaria COX1 gene in crocodile monitors from Thailand. The results from the molecular analysis indicate that the evolutionary distance between the *Hepatozoon* sp. 18S rRNA gene and sheathed microfilaria COX1 gene is greater than the distance between the previously known species, *H. caimani* and *B. timori*, respectively. Consequently, further research focusing on the transmission, interactions between hosts and parasites, and distribution of vectors for these parasites is of utmost importance, particularly in crocodile monitors.

## Materials and methods

### Collection of blood samples and morphological study of the parasites

Two crocodile monitors (*V. salvator*) were captured in Nakhon Pathom province and restrained using a nose pole before being transported to a veterinary hospital. A veterinarian collected peripheral blood samples (n = 2) from the caudal tail vein of the crocodile monitors by using an 18-gauge needle and transferred them into EDTA-treated tubes. The samples were collected in accordance with applicable local guidelines. The blood samples were then submitted to the Vet Central Lab. The blood samples were used in blood smears for microscopic examination. Slides were air-dried, fixed with methanol, and stained with Giemsa^30^. Giemsa-stained thin blood smears were examined microscopically to assess the presence of *Hepatozoon* gamonts and microfilariae, as well as erythrocyte changes caused by the presence of parasites. To examine the intraerythrocytic parasite stages, digital images were obtained and measured using an Olympus CX31 biological microscope (Olympus, Japan) at 100× magnification. The measurements, in micrometers (µm), included the length and width of the parasite, with corresponding mean and standard deviation values (mean ± standard deviation). The remaining EDTA blood sample was preserved at − 20 °C for subsequent molecular analysis.

### DNA extraction, amplification, and sequencing

The collected blood samples were used for DNA extraction. DNA samples were extracted using a genomic DNA blood kit (NucleoSpin^®^ Blood, MACHEREY-NAGEL, Germany). This process was carried out according to the manufacturer’s instructions. Initially, the detection of *Hepatozoon* sp. DNA was achieved using primers targeting part of the 18S rRNA gene, namely HepF300, and HepR900. Positive samples then used the primers HEMO1 and HEMO2 to amplify a partially overlapping fragment of the 18S rRNA gene to obtain a longer gene portion, as shown in Table 1. Microfilariae were detected in blood smears, and three pairs of primers were used to taxonomically identify these parasites: the 18S rRNA, COX1, and 12S rRNA genes, as shown in Table 1. The amplification conditions involved 20 μL PCR reactions, containing DNA template (2 μL), 1X Gotaq^®^ Green Master Mix (Promega, USA), forward and reverse primers (0.2 mM each), and nuclease-free water, and the reaction was performed in a thermal cycler (BIOER technology, China). A positive control for *Hepatozoon* sp. and microfilariae DNA was obtained from a naturally infected dog. Nuclease-free water was used as a negative control. The PCR products were stained with RedSafe™ Nucleic Acid Staining Solution (INtRON Biotechnology, Korea) and analyzed via gel electrophoresis using 1% agarose gels. A 100 bp DNA ladder (SibEnzyme^®^, Russia) was used as the standard for determining the molecular mass of the PCR products. The reaction products were purified using a PCR clean-up gel extraction kit (NucleoSpin^®^ Gel and PCR Clean-up, MACHEREYNAGEL, Germany). Purified amplified DNA fragments were submitted for sequencing using Barcode Taq (BT) sequencing and used for subsequent phylogenetic and haplotype diversity analyses.

### Phylogenetic analysis

Phylogenetic reconstructions were based on the DNA sequence alignment of positive samples. Comparisons with sequences deposited in GenBank used the nucleotide BLAST. The sequences were aligned with sequences published in GenBank using the Clustal W algorithm, available in the MEGA software, version 11.0.13^31^. Phylogenetic relationships were inferred using the Maximum Likelihood (ML) and the Bayesian inference (BI) methods in MrBayes, version 3.1.2^32^. The reliability of inferred phylogenetic relationships was evaluated by the statistical calculation of 1000 replicates using the bootstrapping method^33^. A Bayesian Markov Chain Monte Carlo analysis was conducted with four Markov chains (three heated chains and one cold) for 50,000,000 generations, with the trees sampled every 1000 generations. The first 50% of the trees were discarded and the remaining samples were used to construct a Bayesian consensus tree and to infer the posterior probability. Genetic distances were assessed using distance matrices under the assumption of pairwise-distance^34^ and using the Kimura 2-parameter method^35^. Similarities were evaluated using the sequence identity matrix tool in the BioEdit program, version 7.0.5.3^36^.

### Haplotype diversity analysis

The DNA polymorphisms and haplotype information of *Hepatozoon* sp. and microfilariae sequences were determined using the DnaSP software, version 5.10.01^37^. Haplotype networks were established using the TCS network tool in the Population Analysis with Reticulate Trees (PopART) software^38,39^.

### Ethical approval

This research project was approved by the Biosafety Committee of Chulalongkorn University, Faculty of Veterinary Science (IBC 2231037). The authors would like to confirm that the samples were collected in accordance with applicable local guidelines.

### Supplementary Information

Supplementary Information.

## Data Availability

The original contributions presented in the study are included in the article/Supplementary material, further inquiries can be directed to the corresponding author.

## References

[1] Böhme W (2003). Checklist of the living monitor lizards of the world (family Varanidae). Zool. Verh..

[2] Vidal N (2012). Molecular evidence for an Asian origin of monitor lizards followed by tertiary dispersals to Africa and Australasia. Biol. Lett..

[3] Lauprasert K, Thirakhupt K (2001). Species diversity, distribution and proposed status of monitor lizards (family Varanidae) in southern Thailand. Nat. Hist. J. Chulalongkorn Univ..

[4] Cota M, Chan-Ard T, Mekchai S, Laoteaw S (2008). Geographical distribution, instinctive feeding behavior and report of nocturnal activity of *Varanus dumerilii* in Thailand. Biawak.

[5] Koch A, Ziegler T, Böhme W, Arida E, Auliya M (2013). Pressing problems: distribution, threats, and conservation status of the monitor lizards (Varanidae: *Varanus* spp.) of Southeast Asia and the Indo-Australian Archipelago. Herpetol. Conserv. Biol..

[6] Joshi M, Das SK, Sarma K (2021). Taxonomy, population status and ecology of Indian desert monitor lizard *Varanus griseus koniecznyi* Mertens 1954 in the Thar desert of Rajasthan. Saudi J. Biol. Sci..

[7] Cota M (2009). Study and conservation of varanids of Thailand: Past achievements and future challenges. J. Wildl. Thailand.

[8] Vilcins IM, Ujvari B, Old JM, Deane E (2009). Molecular and morphological description of a *Hepatozoon* species in reptiles and their ticks in the Northern Territory, Australia. J. Parasitol..

[9] Rataj AV, Lindtner-Knific R, Vlahović K, Mavri U, Dovč A (2011). Parasites in pet reptiles. Acta Vet. Scand..

[10] Doornbos K (2013). *Rickettsia* sp. closely related to *Rickettsia raoultii* (Rickettsiales: Rickettsiaceae) in an *Amblyomma helvolum* (Acarina: Ixodidae) tick from a *Varanus salvator* (Squamata: Varanidae) in Thailand. J. Med. Entomol..

[11] Enabulele EE, Ozemoka HJ, Awharitoma AO, Aisien MSO (2013). Parasitic infections of the African dwarf crocodile (*Osteolaemus tetraspis*) and the ornate Nile monitor (*Varanus ornatus*) from Nigeria. Acta Parasitol..

[12] Cook CA, Netherlands EC, Smit NJ (2016). Redescription, molecular characterization and taxonomic re-evaluation of a unique African monitor lizard haemogregarine *Karyolysus paradoxa* (Dias, 1954) n. comb (Karyolysidae). Parasites Vectors.

[13] Calil PR (2017). Hemogregarine parasites in wild captive animals, a broad study in São Paulo Zoo. J. Entomol. Zool. Stud..

[14] Mendoza-Roldan JA (2021). Molecular detection of vector-borne agents in ectoparasites and reptiles from Brazil. Ticks Tick-borne Dis..

[15] Jameie F, Nasiri V, Paykari H (2023). Morphological detection and molecular characterization of *Hepatozoon* spp. from venomous terrestrial snakes in Iran. Exp. Parasitol..

[16] Wicks RM (2006). Morphological and molecular characteristics of a species of *Hepatozoon* Miller, 1908 (Apicomplexa: Adeleorina) from the blood of *Isoodon obesulus* (Marsupialia: Peramelidae) in Western Australia. Syst. Parasitol..

[17] Salakij C (2014). Quantitative and qualitative morphologic, cytochemical, and ultrastructural characteristics of blood cells in captive Asian water monitors. Vet. Clin. Pathol..

[18] Moço TC (2012). Morphological, morphometric and molecular characterization of *Hepatozoon* spp. Apicomplexa, Hepatozoidae) from naturally infected *Caudisona durissa terrifica*. Parasitol. Res..

[19] Tomé B, Maia JPMC, Harris DJ (2012). *Hepatozoon* infection prevalence in four snakes genera: influence of diet, prey parasitemia levels, or parasite type?. J. Parasitol..

[20] Maia JP, Crottini A, Harris DJ (2014). Microscopic and molecular characterization of *Hepatozoon domerguei* (Apicomplexa) and *Foleyella furcata* (Nematoda) in wild endemic reptiles from Madagascar. Parasite.

[21] Ujvari B, Madsen T, Olsson M (2004). High prevalence of *Hepatozoon* spp. (Apicomplexa, Hepatozoidae) infection in water pythons (*Liasis fuscus*) from tropical Australia. J. Parasitol..

[22] Perkins SL, Keller AK (2001). Phylogeny of nuclear small subunit rRNA genes of hemogregarines amplified with specific primers. J. Parasitol..

[23] Harris DJ, Maia JP, Perera A (2011). Molecular characterization of *Hepatozoon* species in reptiles from the Seychelles. J. Parasitol..

[24] Maia JPMC, Harris DJ, Perera A (2011). Molecular survey of *Hepatozoon* species in lizards from North Africa. J. Parasitol..

[25] Mathew JS (2000). Phylogenetic relationships of *Hepatozoon* (Apicomplexa, Adeleina) based on molecular, morphologic, and life-cycle characters. J. Parasitol..

[26] O’Dwyer LH (2013). Description of three new species of *Hepatozoon* (Apicomplexa, Hepatozoidae) from rattlesnakes (*Crotalus durissus terrificus*) based on molecular, morphometric and morphologic characters. Exp. Parasitol..

[27] Telford SR, Butler JF, Telford RS (2005). *Hepatozoon polytopis* n. sp. parasitic in two genera and species of colubrid snakes in Southern Florida. J. Parasitol..

[28] Barta JR, Ogedengbe JD, Martin DS, Smith TG (2012). Phylogenetic position of the adeleorinid coccidia (Myzozoa, Apicomplexa, Coccidia, Eucoccidiorida, Adeleorina) inferred using 18S rDNA sequences. J. Euk. Microbiol..

[29] Uni S (2017). Morphological and molecular characteristics of *Malayfilaria sofiani* Uni, Mat Udin & Takaoka n. g., n. sp. (Nematoda: Filarioidea) from the common treeshrew *Tupaia glis* Diard & Duvaucel (Mammalia: Scandentia) in Peninsular Malaysia. Parasites Vectors.

[30] Rosenblatt JE (2009). Laboratory diagnosis of infections due to blood and tissue parasites. Clin. Infect. Dis..

[31] Saitou N, Nei M (1987). The neighbor-joining method: A new method for reconstructing phylogenetic trees. Mol. Biol. Evol..

[32] Huelsenbeck JP, Ronquist F (2001). MRBAYES: Bayesian inference of phylogenetic trees. Bioinformatics.

[33] Felsenstein J (1985). Phylogenies and the comparative method. Am. Nat..

[34] Nei, M. & Kumar, S. *Molecular Evolution and Phylogenetics*. (Oxford University Press, 2000).

[35] Kimura M (1980). A simple method for estimating evolutionary rates of base substitutions through comparative studies of nucleotide sequences. J. Mol. Evol..

[36] Hall TA (1999). BioEdit: A user-friendly biological sequence alignment editor and analysis program for windows 95/98/NT. Nucleic Acids Symp. Ser..

[37] Librado P, Rozas J (2009). DnaSP v5: A software for comprehensive analysis of DNA polymorphism. Bioinformatics.

[38] Clement, M., Snell, Q., Walker, P., Posada, D. & Crandall, K. TCS: Estimating gene genealogies, in *Parallel and Distributed Processing Symposium. International Proceedings*, Vol. 2, 184 (2002). 10.1109/IPDPS.2002.1016585.

[39] Leigh JW, Bryant D (2015). PopART: Full-feature software for haplotype network construction. Methods Ecol. Evol..

[40] Laidoudi Y, Ringot D, Watier-Grillot S, Davoust B, Mediannikov O (2019). Cardiac and subcutaneous canine dirofilariosis outbreak in a kennel in central France. Parasites.

[41] Laidoudi Y (2019). Development of a multiplexed qPCRs-based approach for the diagnosis of *Dirofilaria immitis*, *D. repens*, *Acanthocheilonema reconditum*. Parasites Vectors Dev..

[42] Laidoudi Y (2020). Detection of canine vector-borne filariasis and their *Wolbachia* endosymbionts in French Guiana. Microorganisms.

