# Supplementary material for: First molecular detection and genetic diversity of Hepatozoon sp. (Apicomplexa) and Brugia sp. (Nematoda) in a crocodile monitor in Nakhon Pathom, Thailand
Source: Sci Rep. 2024 Feb 12;14:3526. doi: 10.1038/s41598-024-54276-6 (PMC10861490; doi:10.1038/s41598-024-54276-6)
Supplement: Supplementary file 1 — Supplementary Information. [file 41598_2024_54276_MOESM1_ESM.docx]

###
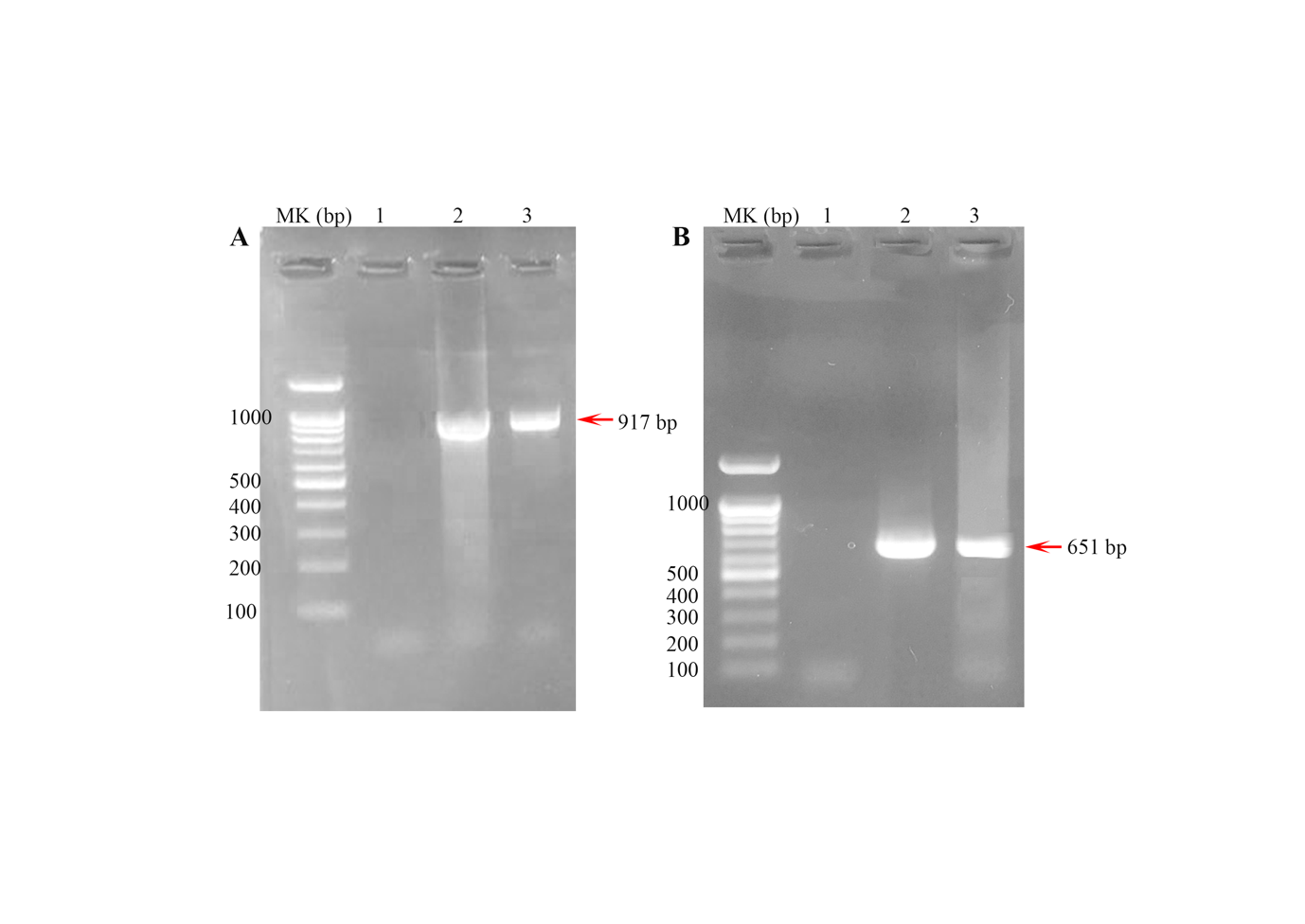


### Supplementary Figure 1. PCR products of *Hepatozoon* 18S rRNA and sheathed microfilaria COX1 genes from crocodile monitors in Thailand demonstrating a 917 base pair (bp) fragment of the 18S rRNA gene (A), which was the negative control (Lane 1). The *Hepatozoon* sp. DNA positive control was obtained from a naturally infected dog (Lane 2). Test DNA was obtained from a crocodile monitor (Lane 3), and a 615 bp fragment of the COX1 gene (B) acted as the negative control (Lane 1). A microfilariae DNA positive control was obtained from a naturally infected dog (Lane 2), and test DNA was obtained from a crocodile monitor (Lane 3). The molecular size standard is a 1500 bp ladder.
